# Integrating Human-Centered Design Methods Into a Health Promotion Project: Supplemental Nutrition Assistance Program Education Case Study for Intervention Design

**DOI:** 10.2196/37515

**Published:** 2023-04-21

**Authors:** Elizabeth Chen, Jared Bishop, Lindsay Guge Cozon, Eduardo Hernandez, Claire Sadeghzadeh, Megan Bradley, Tracy Dearth-Wesley, Molly De Marco

**Affiliations:** 1 Department of Health Behavior Gillings School of Global Public Health The University of North Carolina at Chapel Hill Chapel Hill, NC United States; 2 Center for Health Promotion and Disease Prevention The University of North Carolina at Chapel Hill Chapel Hill, NC United States; 3 Share Our Strength Washington, DC United States; 4 Department of Nutrition Gillings School of Global Public Health The University of North Carolina at Chapel Hill Chapel Hill, NC United States

**Keywords:** human-centered design, design thinking, program development, stakeholder engagement, nutrition, parenting, children, pediatrics

## Abstract

**Background:**

Human-centered design, or design thinking, offers an extensive toolkit of methods and strategies for user-centered engagement that lends itself well to intervention development and implementation. These methods can be applied to the fields of public health and medicine to design interventions that may be more feasible and viable in real-world contexts than those developed with different methods.

**Objective:**

The design team aimed to develop approaches to building food skills among caregivers of children aged 0-5 years who are eligible for a federal food assistance program while they were in the grocery store.

**Methods:**

They applied 3 specific human-centered design methods—Extremes and Mainstreams, Journey Mapping, and Co-Creation Sessions—to collaboratively develop intervention approaches to enhance Supplemental Nutrition Assistance Program Education (SNAP-Ed) reach and impact across food retail settings. Extremes and Mainstreams is a specific kind of purposive sampling that selects individuals based on characteristics beyond demographics. Journey Mapping is a visual tool that asks individuals to identify key moments and decision points during an experience. Co-Creation Sessions are choreographed opportunities for individuals to explicitly contribute to the design of a solution alongside research or design team members.

**Results:**

Ten caregivers with diverse lived experiences were selected to participate in remote design thinking workshops and create individual journey maps to depict their grocery store experiences. Common happy points and pain points were identified. Nine stakeholders, including caregivers, SNAP-Ed staff, and grocery store dieticians, cocreated 2 potential intervention approaches informed by caregivers’ experiences and needs: a rewards program and a meal box option.

**Conclusions:**

These 3 human-centered design methods led to a meaningful co-design process where proposed interventions aligned with caregivers’ wants and needs. This case study provides other public health practitioners with specific examples of how to use these methods in program development and stakeholder engagement as well as lessons learned when adapting these methods to remote settings.

## Introduction

In the development and implementation of public health and medical interventions, clinicians and researchers must consider the needs and desires of the unique communities in which they operate. Human-centered design (HCD), or design thinking, offers an extensive toolkit of methods and strategies for user-centered engagement that lends itself well to intervention development and implementation [1]. Although HCD has been applied mostly in the business and engineering sectors, there are a growing number of projects that integrate HCD into education [2-6], medicine [7-9], public policy [10,11], and other areas of research [12]. Public health projects that use an HCD approach can better ensure that an intervention’s aims are well aligned with the needs and goals of the intended audience and consider solutions’ feasibility and viability early on [1,13]. Public health researchers are starting to apply HCD methods alongside community-based participatory research (CBPR) [13] and implementation science [14] methods to address topics including chronic disease prevention [15], patient emotional well-being [16], and health disparities [17]. For example, Kia-Keating et al [17] combined CBPR and HCD methods to engage Latine youth in community conversations and action planning to address violence-related health disparities. This research team incorporated HCD methods of conversation starters [18] and storyboards [19] into their research process. HCD methods can be used to better understand communities and context as well as to develop or adapt interventions [20].

The design team aimed to develop approaches to building food skills among Supplemental Nutrition Assistance Program (SNAP)–eligible caregivers of children aged 0-5 years for SNAP Education (SNAP-Ed) Implementing Agencies (IAs) to deploy within food retail settings. Food retail settings are embedded within complex systems that include a diverse group of stakeholders, wide-ranging values, and varying resources that yield facilitators and barriers to successful implementation. HCD provides an opportunity to build food skills that center SNAP-eligible caregivers’ needs in these environments while also addressing key system stakeholders’ ideas and concerns for implementation. SNAP-Ed IAs implement a range of direct education (eg, cooking and nutrition classes for adults); social marketing (eg, campaigns to increase water consumption); and policy, systems, and environmental change strategies (eg, multisectoral collaboration to improve conditions to be physically active in communities through programs like Safe Streets, improving lighting and building walking trail) to encourage and support healthy living for SNAP-eligible households and low-income communities. More on SNAP-Ed approaches can be found in the SNAP-Ed Toolkit [21].

This paper details 3 specific HCD methods—Extremes and Mainstreams, Journey Mapping, and Co-Creation Sessions—that the design team applied to enhance SNAP-Ed reach and impact. An HCD approach was chosen by the design team because Share Our Strength’s Cooking Matters (the funder) wanted to ensure that the design team centered the voice of parents and caregivers in the research and findings. This paper was written to describe the methods in detail so other public health professionals can replicate or adapt these methods to strengthen user engagement, especially in the intervention design process. The paper also offers specific guidance on adapting HCD methods to a remote environment and shares lessons learned and recommendations for -creating a safe, welcoming environment, building off the team’s experience by conducting these activities during the COVID-19 pandemic. Lastly, the paper follows detailed reporting guidelines of health research involving design put forth by Bazzano et al [20]. To see our table of included elements, see Multimedia Appendix 1.

## Methods

### Approach

IDEO, a world-renowned design company, has an accessible HCD process that is organized into 3 distinct phases: Inspiration, Ideation, and Implementation [22]. Although the design team used multiple HCD methods throughout the project as detailed in Multimedia Appendix 2, this paper focused on 3 HCD methods because of their relevance to a wide range of public health projects: Extremes and Mainstreams (Inspiration), Journey Mapping (Ideation), and Co-Creation Sessions (Ideation and Implementation). For additional information about the methods included in Multimedia Appendix 2, please consult IDEO.org’s *Field Guide* [22].

### Overview

The design team consisted of 3 facilitators and 1 research lead. The research lead was an evaluation specialist with training in health behavior, design thinking, and qualitative and participatory research methods Additionally, the research lead had 10 years of experience working with local, state, and federal programs serving families eligible for nutrition assistance programs in a variety of community-based settings, including grocery stores. The facilitators and research lead worked collaboratively to design activities and synthesize and analyze data between sessions. All 3 facilitators had previous experience with design thinking methods and mindsets and were students enrolled in master’s of public health or social work programs. One facilitator was a caregiver of young children, and one facilitator was a native Spanish speaker. The broader research team also included an assistant professor in the department of nutrition, a nutritional epidemiologist, an assistant professor in health behavior with formal HCD training from IDEO and a university-level design thinking leadership role, and a senior leader from the national organization funding this project with expertise in food skills who regularly engages with SNAP-Ed IAs. All team members were based in North Carolina except for the senior leader who was based in Washington DC. The 3 facilitators led the sessions and managed communications with the participants. Each session had at least 1 facilitator and 1 cofacilitator. The cofacilitator helped set up breakout rooms, supported participants with technical issues, kept track of time, and monitored the chat.

The design team recruited SNAP-eligible caregivers to participate in remote design thinking workshops. Together, they brainstormed initial ideas for strategies to impart food skills education in this setting. After these sessions, the design team hosted a series of cocreation sessions with caregivers, SNAP-Ed IAs, and key stakeholders in the food retail setting to further conceptualize and test these ideas. The resulting product was an intervention guide, *Using Human-Centered Design to Test and Implement Food Retail-Level Interventions to Promote Healthy Food Choices Among Caregivers of Young Children*, detailing the HCD methods used and presenting 2 unique interventions for promoting healthy food choices in the food retail environment.

### Ethical Considerations

The research protocol was reviewed and deemed exempt by the University of North Carolina at Chapel Hill’s institutional review board (IRB 20-1649).

The design team contacted eligible participants (identified through a survey) by email in their primary language (Spanish or English) to invite them to participate, detailed the time commitment and activities they would be participating in, explained potential risks and benefits, and invited any questions. A consent form was attached to the invitation email. The design team received verbal consent from people who joined the sessions.

### Extremes and Mainstreams

In June 2020, the design team recruited a diverse group of 10 users from their intended audience—caregivers of young children (aged 0-5 years) who were eligible for SNAP. IDEO’s Extremes and Mainstreams method [23] challenged the typical public health recruitment strategies—“high-risk” or “population” approaches—and encouraged the team to expand how team members understood what it means for a sample to be “diverse.” Although a “high-risk” approach typically requires recruiting those who are at greatest risk for a particular health behavior or outcome [24,25] and a “population” approach typically requires recruiting those within the middle of a risk distribution curve [26,27], an HCD approach hinges on the premise that an idea that is designed for “extremes”—users on opposite ends of a variety of spectrums—will work for those in the middle of the spectrum too, the “mainstreams.” Collaborating with extreme users can creatively expose a design team to other ideas, solutions, and design opportunities [23]. The use of the Extremes and Mainstreams method led the team to explore a broader spectrum of caregivers based on lived experiences, preferences, behaviors, or other characteristics that are less often collected.

The members of the design team conducted 3 key informant interviews with SNAP-Ed IAs and public health practitioners who use design thinking in their work to learn about the feasibility of engaging caregivers virtually, strategies for reaching caregivers digitally (ie, recruitment channels), and limitations or challenges for adapting the work to a remote format. The design team also conducted a literature review to identify key characteristics they may want to select for when engaging the primary audience (eg, perceptions of SNAP-Ed, readiness for change, number of children, type of childcare used, or participation in supplemental nutrition and safety net programs). The team developed a web-based survey to gauge interest, screen eligible caregivers, and capture responses to questions about these predefined characteristics from eligible caregivers. Then they partnered with SNAP-Ed IAs in 5 priority states (North Carolina, Colorado, Oklahoma, Massachusetts, and California) to distribute English and Spanish versions of the survey. In order to select these 5 states, the design team started with the 13 states identified by Share Our Strength’s Cooking Matters as priority states based on (1) potential reach of their priority population (ie, the Cooking Matters universe) and (2) existing needs based on a review of WIC (Special Supplemental Nutrition Program for Women, Infants, and Children) coverage, as well as high rates of chronic disease and obesity and low reported rates of positive health behaviors. They used sampling on the extremes (a type of purposive sampling) to select 5 states.

The team reviewed the data and developed a “typical” persona by identifying average responses, which allowed them to identify unique or differing responses (ie, the extremes). For example, the design team was interested in inviting participants with varied perceptions of food skills education (eg, people who believed, did not believe, or were neutral around food skills education being impactful or a beneficial use of time). In theory, representation from all perceptions would help the team design an attractive intervention for a wide audience. From these data, a diverse group of mainstream and extreme users were selected to participate in the project.

### Journey Mapping

The design team engaged the selected caregivers in four 2-hour long remote HCD workshops via Zoom to learn about their needs, desires, and experiences interacting with the food retail setting. These workshops took place from August 2020 through September 2020. Participating caregivers were compensated US $50 per hour for their time through VISA debit cards. In these biweekly Ideation phase workshops, the team engaged in Journey Mapping [28] with caregivers to capture and visualize their user experience through the grocery store from beginning to end. Public health researchers often spend a lot of time focusing on designing products, services, or interventions by themselves with less attention to what happens before and after a user interacts with their solution. Journey Mapping is a tool that helps others understand the larger journey for which they are designing and asks users to identify critical moments in that journey.

Although the design team intended to have caregivers use the web-based digital tool Miro to create their individual journey maps, caregivers struggled with the digital tool in earlier sessions, so the team adapted the instructions and asked caregivers to share the steps they complete before, during, and after a grocery store visit by completing a short web-based survey. Before the session, the team created simple visual maps in Google slides with their survey responses to help guide discussions. During the session, team members asked each caregiver to present their individual map and invited other caregivers to ask questions and update their own journey maps as others shared. Team members also asked caregivers to identify steps that they considered “happy points” and “pain points.” Happy points were defined as moments that brought the caregivers joy or happiness. Pain points were defined as moments that were tiresome, uncomfortable, or time-consuming for caregivers. By including happy and pain points directly on the journey map, the team gained valuable insight into the instances that caregivers enjoyed and disliked, what made them enjoyable or not enjoyable, and where and when they may be open to food skills education in their journey.

The team used thematic content analysis to identify common experiences across participants. The team used multiple analytic processes including affinity mapping and matrices between sessions to organize and identify themes and common steps to develop a consolidated common journey across participants. For example, the checkout area was a common pain point for participants because of the number of tasks they must finish to complete their visit (eg, check budgets, check lists, and provide multiple forms of payment) and the mental fatigue experience after shopping. The team shared this map with participants to receive feedback, which was integrated into the final product. The individual and consolidated journey maps served as the foundation for future brainstorming sessions to facilitate the generation of ideas for interventions.

### Co-Creation Sessions

The purpose of a Co-Creation Session is to convene a group of stakeholders to directly involve them in the design process [29]. The design team convened 3 caregivers from the journey mapping sessions, 3 SNAP-Ed leaders, and 3 stakeholders with experience in the food retail setting (2 supermarket registered dietitians and 1 nutrition and culinary consultant working with grocery store chains). The team facilitated four 2-hour-long cocreation sessions from December 2020 to January 2021, with the goal to develop 2 ideas that emerged from the initial caregiver workshops. SNAP-Ed IAs helped the design team consider SNAP-Ed program constraints and needs (eg, allowable costs), and the food retail stakeholders helped the design team understand the logistical and relational considerations when working in grocery stores (eg, equipment and key drivers). All perspectives were critical to the success of developing a desirable intervention for caregivers that could be delivered by SNAP-Ed IAs in partnership with grocery stores.

Before the cocreation sessions began, the design team discussed real and perceived power dynamics within the group and brainstormed potential ways to mitigate and manage them to ensure that caregivers were centered throughout the process. These power dynamics could arise in multiple scenarios, including between caregivers and SNAP-Ed IAs, between SNAP-Ed IAs and food retail stakeholders, and between the design team and all cocreation participants. The design team adopted strategies to be mindful of and hold power dynamics within the group, including (1) establishing group norms to recognize and celebrate each person’s unique contributions to the design solution (ie, no one knows everything, together we know a lot), (2) starting each session by revisiting caregiver insights and coming back to these during critical decision-making points to ensure decisions aligned with caregivers’ needs and desires, and (3) including a reflection question for facilitators to respond to after each session on any power dynamics. The reflection question included prompts on the frequency and quality of participation within the group and ideas to improve facilitation during the next session.

During the first session, team members asked the cocreation participants to reflect on key insights and initial intervention ideas generated during the caregiver sessions. Guiding questions for this discussion included “What are some opportunities or possibilities for these ideas to come to life?” and “What insights do you have from past experiences or projects that can be related to these interventions?” Then each co-creation participant was asked, via a Zoom poll, to select their top 2 favorite interventions that the team should explore in future phases of this project. The 2 intervention ideas that were selected by the co-creation participants included a rewards program and a meal box containing ingredients and recipe cards.

During session 2, the design team divided the cocreation participants into 2 groups via Zoom breakout rooms so one group could work collaboratively on the meal box and the other on the rewards program. Each participant then sketched a prototype (ie, an initial draft) of their assigned intervention idea using paper and pen. They took pictures of their prototype sketches using their smartphones and emailed them to the design team. After the team reviewed the prototypes, they identified common elements of the individual prototypes and presented a synthesized prototype for each of the 2 intervention ideas. Then they shared back the synthesized prototypes with participants via email and included a Qualtrics survey to gather feedback before the third session. The design team then hosted 2 additional cocreation sessions to further refine the 2 intervention ideas. See Multimedia Appendix 2 to learn about additional HCD methods used in additional sessions.

After the 4 sessions, the design team continued to package the intervention ideas into a step-by-step guide for SNAP-Ed IAs, integrating key insights and feedback about SNAP-Ed appropriateness, implementation options based on the grocery store partner, and caregiver needs. The design team hosted 1 additional cocreation session with food retail stakeholders to gather feedback on operations and logistics for the 2 proposed interventions given their experience and knowledge of the retail setting. The final guide presented the 2 intervention ideas with several options for a variety of local contexts and a testing template, which can help SNAP-Ed IAs move these ideas into action in the communities they serve.

## Results

### Extremes and Mainstreams

The team received 66 responses to the eligibility screener, reviewed survey results, and discussed them to identify a diverse group of caregivers. The team then selected 10 caregivers to participate in the remote HCD workshops.

Table 1 describes the characteristics of the caregivers selected after considering the Extremes and Mainstreams characteristics. Key characteristics that helped with sample selection included age, employment status, participation in supplemental nutrition and safety net programs, and type of childcare used. Sampling across the extremes helped the team recruit participants with vastly different experiences beyond demographics. For example, this sampling method helped the team recruit 1 caregiver who was in a domestic partnership, a student, self-employed, and raising 3 children (1 in high school and 2 younger than 5 years). Another caregiver was a single parent living in a women’s and children’s housing program with her 3 boys (1 middle school, 1 elementary school, and a 1-year-old) and worked as a chief grocery store deli clerk.

**Table 1:** Characteristics of caregivers participating in SNAP-Ed^a^ design thinking sessions (N=10).

Characteristic		Value, n (%)
**Age group (years)**		
	18-29	1 (10)
	30-39	8 (80)
	40-49	1 (10)
**Gender identity**		
	Woman	10 (100)
**Ethnicity**		
	Hispanic or Latino/a/x	2 (20)
	Not Hispanic or Latino/a/x	8 (80)
**Race**		
	American Indian or Alaskan Native	1 (10)
	Black or African American	3 (30)
	White	6 (60)
**Highest level of education completed**		
	High school diploma or GED^b^	2 (20)
	Vocational, technical, or trade school	1 (10)
	Some college	2 (20)
	Associate’s degree	1 (10)
	Bachelor’s degree	4 (40)
**Employment^c^**		
	Employed (part-time)	4 (40)
	Employed (full-time)	1 (10)
	Self-employed (part-time)	2 (20)
	Stay-at-home caregiver	1 (10)
	Student	4 (40)
	Retired	1 (10)
**Marital status**		
	Married or domestic partnership	5 (50)
	Separated	2 (20)
	Single, never married	3 (30)
**Participation in the Supplemental Nutrition and Safety Net Programs^c^**		
	Food pantry	7 (70)
	Free or reduced-price school meals	6 (60)
	Free summer meals	5 (50)
	Head Start	3 (30)
	Medicaid	7 (70)
	SNAP or Electronic Benefit Transfer (ie, EBT)	7 (70)
	Women, Infants, and Children (ie, WIC)	9 (90)
**Number of children <6 years old at home**		
	1 child	6 (60)
	2 children	3 (30)
	3 children	1 (10)
**Type of childcare**		
	At-home only	3 (30)
	At-home and center-based childcare	1 (10)
	At-home, center-based childcare, home-based childcare, and friend or family provides childcare	1 (10)
	At-home and friend or family provides childcare	1 (10)
	Center-based childcare only	1 (10)
	At-home, friend or family provides childcare, and partner or spouse provides childcare	1 (10)
	Center-based childcare, and friend or family provides childcare	1 (10)
	Home-based childcare, and partner or spouse provides childcare	1 (10)
**State**		
	California	2 (20)
	Colorado	1 (10)
	Massachusetts	2 (20)
	North Carolina	2 (20)
	Oklahoma	3 (30)
**Type of neighborhood**		
	Rural	1 (10)
	Suburban	6 (60)
	Urban	3 (30)
**Language preferred for design thinking sessions**		
	English	10 (100)
**Technology preferred for design thinking sessions**		
	Computer or laptop	3 (30)
	Mobile phone	7 (70)

^a^SNAP-Ed: Supplemental Nutrition Assistance Program Education.

^b^GED: General Education Development.

^c^Caregivers could select multiple options for Employment and Supplemental Nutrition and Safety Programs. Numbers and percentages reflect participation in individual types of work and programs.

### Journey Mapping

**Figure 1:**
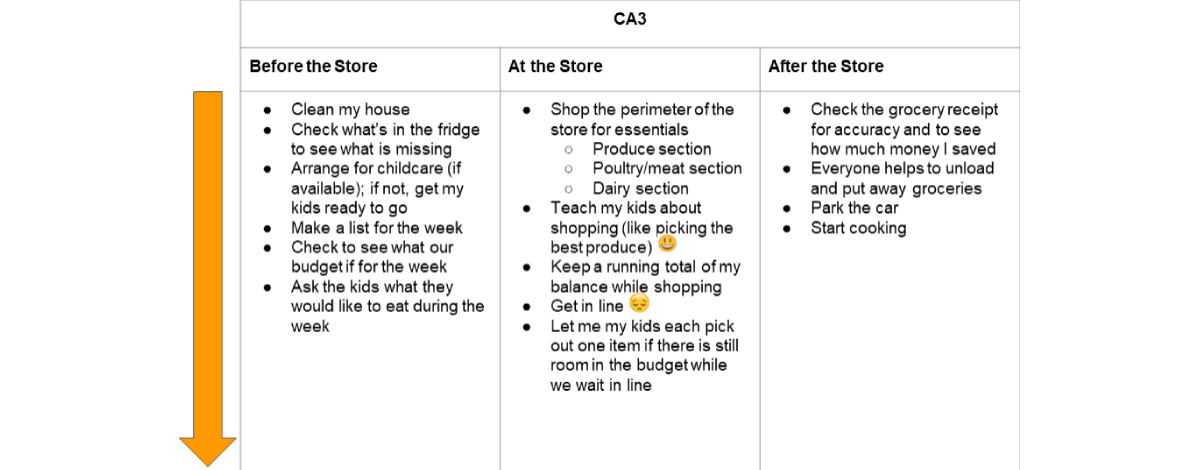
Journey map from a caregiver in Colorado. CA3: participant ID number.

Each of the 10 caregivers created an individual journey map (see Figure 1 for an example of an individual caregiver journey map).

**Figure 2:**
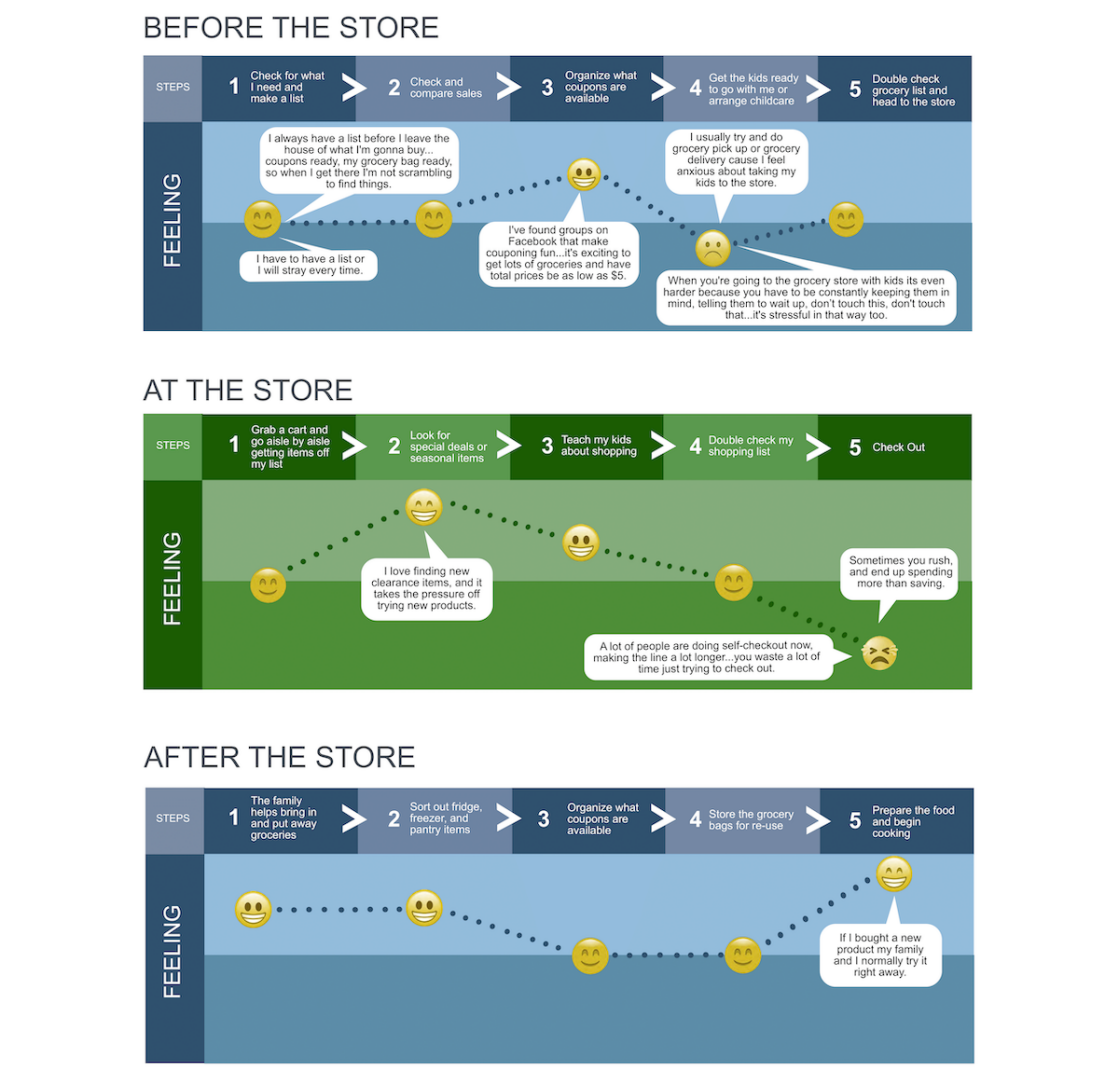
Consolidated journey map with happy and pain points.

The consolidated journey map is pictured in Figure 2. Two common pain points included having to keep their children entertained and calm during the shopping experience and feeling overwhelmed or cognitively burdened at checkout. Two common happy points at the store were finding clearance items and teaching their kids about healthy food shopping and budgeting. The experiences in the individual journey maps led to insights into what types of food skills education caregivers would like and when they would be open to receiving it. For example, caregivers unanimously disliked waiting in line at the grocery store and identified this location at the grocery store as a stressor, so this time would not be an appropriate time to approach caregivers during their shopping experience. The team also learned that caregivers wanted food skills education opportunities to also engage their children throughout the process.

### Co-Creation Sessions

**Figure 3:**
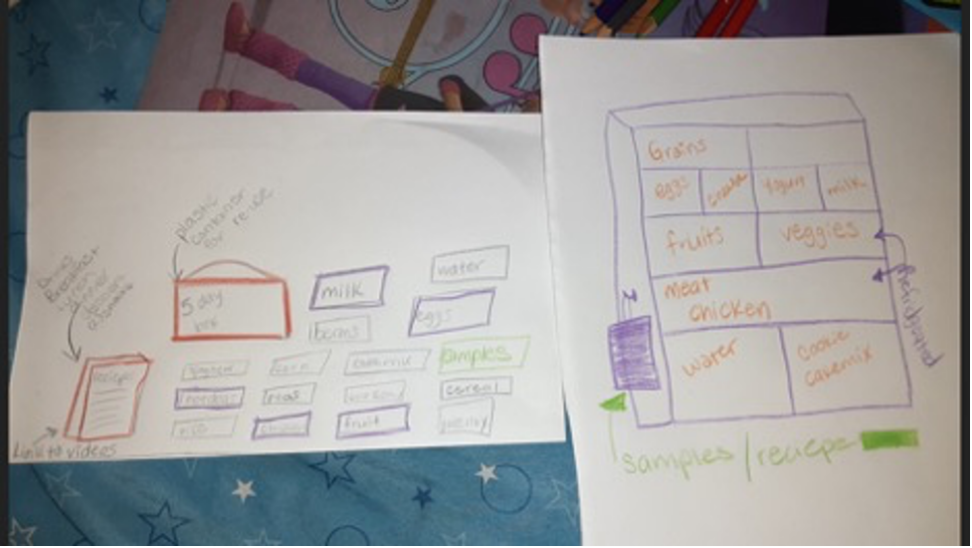
Meal box prototype sketches from a co-creation session participant.

Figure 3 includes sample prototype sketches from a cocreation session participant depicting her ideas for a meal box intervention. Common ideas across meal box prototypes drawn by participants included offering ingredients for multiple meals within one box, providing recipe cards with meal box ingredients, incorporating kid-friendly activities or materials in the box, and colocating the meal box components in a central location within the retail store. Results from these cocreation sessions also highlighted the key aspects of the emerging intervention that needed further development, including how the meal boxes would be priced and what types of marketing efforts might be best received by SNAP-eligible caregivers. Additionally, the cocreation sessions helped the team identify outstanding questions and decisions, like who would select the recipes and ingredients in the meal boxes.

The prototyping and brainstorming in these sessions led to the development of a decision tree for SNAP-Ed IAs and food retail partners based on food retail store capacity, staff availability, and other key considerations (Multimedia Appendix 3). This decision tree offered 3 options for meal box intervention: Pre-Packaged Box Pick-Up, One Stop Shop Area (pictured in Figure 3), and List and/or Map of Ingredients.

Initial prototyping also led to the development of other tools for the Food Retail guide, such as an interview guide for SNAP-Ed IAs to use with their food retail partner to navigate the decision tree.

## Discussion

### Principal Findings

Each of the 3 HCD methods detailed above yielded different insights and lessons learned for the project. The use of the Extremes and Mainstreams method resulted in the recruitment of caregivers who were diverse beyond their geography and demographic information. Conducting key informant interviews and a literature review were critical in developing the survey. The intentionally developed web-based screener survey was important as the design team used the data collected to make decisions about recruitment.

The design team found that journey maps were the most valuable HCD method to integrate into sessions with caregivers. In designing solutions to health issues, a common failing is to focus only on the solution itself; researchers less often consider the broader experience or context in which solutions are situated. The use of journey maps held the team accountable for taking these key contextual factors and moments into consideration. For the purposes of this project, the design team explicitly considered caregivers’ experiences before, during, and after the intervention. The individual journey maps clarified caregivers’ day-to-day experiences in food retail settings, and the consolidated journey map was beneficial because it elevated shared decision points, moments of delight, and moments of frustration.

The cocreation sessions not only generated several different ideas for an intervention (the meal box idea) but also surfaced the need for food retail partners to have flexibility and autonomy in decision-making. Instead of expecting all food retail partners to implement the same meal box intervention, the design team created a decision tree with 3 distinct options that guided food retail partners to consider their own context (eg, capacity and staff availability) and make a choice for their setting. It was important to appreciate local context and strengthen stakeholder agency rather than offering a one-size-fits-all, top-down approach.

### Adapting HCD for a Remote Environment

Although the HCD methods were designed for in-person engagements [22], the project’s launch coincided with the start of the COVID-19 pandemic in early 2020. In response, the design team transitioned project activities to a web-based format. This transition offered many benefits including (1) recruitment of caregivers from 5 states to participate together; (2) removal of common barriers to participation like transportation or childcare; (3) lessened perceived power dynamics because everyone was on a screen and markers of status (eg, clothes, phone, and cars) were less visible, especially if the use of Zoom background was encouraged; (4) technology like web-based surveys and remote whiteboard platforms decreasing the need for team members to transcribe or document activities; (5) feedback on journey maps and prototypes incorporated in real time using technology; and (6) technology created a sense of anonymity at times, which encouraged a freer flow of ideas.

Although there were many benefits to transitioning to a remote environment, the design team did identify several barriers: (1) participants differed in their comfort level using technology and some had little experience with videoconferencing, (2) some participants were limited by their device type (eg, phone vs laptop) in how they engaged in some remote activities, (3) the team could not rely on typical facilitation skills like reading nonverbal cues, and (4) it was more difficult to build rapport with participants via Zoom as encounters were very time-bound and limited. For those who choose to adapt these HCD methods to a remote environment, the design team recommends that 1 team member be assigned as the dedicated technology person to manage logistics (eg, setting up breakout rooms) and offer troubleshooting assistance before the session. In addition, the design team found success in establishing and reinforcing group norms at the start of each remote session to cultivate a welcoming and supportive environment for sharing, learning, and brainstorming to strengthen rapport.

Lastly, the design team recommends that particular attention be paid to elevating the voices of participants with lower status or power, especially if they are engaging with those with higher status or power. For this project, this meant elevating the “no one knows everything; together we know a lot” norm and introducing caregivers as experts in meetings with other SNAP-Ed IA stakeholders.

### Comparison With Prior Work

Public health researchers have used the journey mapping method or related methods (eg, journey models or customer journeys) in more recent works [27,30,31], especially integrated into qualitative research methods. Similar to the experiences of those on this design team, these additional researchers found that journey maps were effective at identifying potential moments for intervention and highlighting decision-making factors [15,27,30,31].

Although the use of the Extremes and Mainstreams method is not yet well documented in the peer-reviewed literature, it is related to a type of purposive sampling called maximum variation sampling that is used in both qualitative and quantitative research [32,33]. This data collection procedure is based on the principle of maximum diversity, which is an extension of the statistical principle of regression toward the mean. Instead of seeking representativeness through equal probability sampling, representativeness is sought by including a broad range of extremes [33]. By deliberately selecting a different selection of people, their aggregate answers will be close to the average [33]. Some who use maximum variation sampling choose factors and dimensions that are related to demographics like urbanicity, gender identity, education level, family income level, and other indicators [33]. Others have a broader definition of factors and dimensions and expect that the maximum variation sampling to be an iterative process as researchers will not know what *variation* looks like at the onset [34]. IDEO’s Extremes and Mainstreams method does not intend to capture a representative or “average” set of participants but expects an iterative sampling process like Palinkas et al [34] describe. In addition, the dimensions and factors used in Extremes and Mainstreams often go beyond demographics and descriptors to capture knowledge, attitudes, and behaviors.

Cocreation sessions are not unique to HCD, as there are plenty of examples of their use within other approaches including CBPR to create interventions, policies, and much more [35]. In addition, there are additional approaches like crowdsourcing [36,37] and hackathons [38] that tend to be shorter or one-time engagements, and a new field of social innovation in health research is emerging [39]. Each of these approaches is intended to engage real people in solution design processes. Cocreation sessions as part of HCD are intended to be one method out of many to engage stakeholders, and there are other participatory methods that inform the cocreation session and that are informed by the cocreation session. Therefore, HCD cocreation sessions are distinct from the crowdsourcing and hackathon activities that often are the featured activity in a solution design process.

Although HCD continues to become more popular in public health research, there are critiques and concerns about the approach to note as well. Some of these challenges include (1) developing shallow or obvious ideas, especially if ample time was not allotted for the HCD process or those who were recruited were not proximate to the challenge; (2) teamwork conflicts; (3) a sprint versus a long-term focus; and (4) prioritization of idea creation over implementation or evaluation [4]. The effectiveness of HCD and design thinking methods will depend on the users’ understanding and intent [11]. In addition, it is very time-consuming and resource intensive. As more public health researchers and practitioners apply HCD methods into their work, it is important to describe the rationale, methods, and findings in detail using checklists like the one used to guide the writing for this paper [20]. Researchers must be more transparent about HCD and findings so that others can learn.

### Limitations

Although this study protocol had numerous strengths, there are always limitations to consider. With regard to strengths, this study explicitly incorporated HCD methods that are newly being applied to public health research to inform intervention development for a future SNAP-Ed intervention. This was a novel approach. The design team also implemented the Extremes and Mainstreams, Journey Mapping, and Co-Creation Sessions with fidelity; meaningfully engaged with a diverse set of SNAP-Ed stakeholders; documented their process in detail; and pivoted with success to a remote format for all research activities during the COVID-19 pandemic. In terms of limitations, the design team chose to solely apply IDEO’s version of HCD for this project to maintain consistency and fidelity to one approach. IDEO’s version of HCD was developed for business clients by White men, and there are updated HCD tools from organizations like Creative Reaction Lab [40] and Equity Meets Design [41] that incorporate equity and historical lenses into their approaches. In addition, this study was not designed to measure the effectiveness of this intervention development approach compared with other approaches. Therefore, the design team is not able to comment on whether an HCD approach is better or more effective than others. In addition, the design team did not invite caregivers in early planning conversations or key informant interviews before implementing the remote HCD sessions. These conversations and interviews took place in spring 2020 at the start of the pandemic, and the team did not want to further burden caregivers during a challenging time, but in retrospect, this would have been a better way forward.

### Conclusions

The 3 HCD methods outlined in this case study can be used by public health practitioners, medical professionals, and researchers to strengthen user engagement. Additional research is needed to test these intervention ideas in the real world and to collect evaluation data on their desirability, feasibility, and viability in addition to their effectiveness. Although HCD methods are still new to the public health field, they are tools that help researchers and practitioners consider the needs and desires of the unique communities in which they operate. As health professionals apply HCD methods, they must consider a variety of factors to ensure alignment with project outcomes. Specifically, through this project, the design team found that the use of technology, the consideration of time—both in length of the session and length of the project—and reflection on power dynamics across stakeholders were vital to the successful application of the methods. HCD methods can be applied by teams that are open and flexible in their intervention design and implementation approach as the solution or end product may not be what was originally envisioned. However, this solution will be centered around the user experience, needs, and desires leading to public health or medical interventions with the potential for better implementation outcomes.

## Appendix

Multimedia Appendix 1 Inclusion of detailed reporting elements for reporting of global health research that has used design.

Multimedia Appendix 2 Food, Fitness, and Opportunity Research Collaborative (FFORC) Project Human-Centered Design (HCD) Methods by IDEO HCD Phase.

Multimedia Appendix 3 Meal Box Decision Tree and Intervention Options.

## Abbreviations

CBPR: community-based participatory research
HCD: human-centered design
IA: Implementing Agency
SNAP-Ed: Supplemental Nutrition Assistance Program Education
