# Supplementary material for: Integrating Human-Centered Design Methods Into a Health Promotion Project: Supplemental Nutrition Assistance Program Education Case Study for Intervention Design
Source: JMIR Form Res. 2023 Apr 21;7:e37515. doi: 10.2196/37515 (PMC10163394; doi:10.2196/37515)
Supplement: Multimedia Appendix 1 [file formative_v7i1e37515_app1.docx]

**Multimedia Appendix 1. Inclusion of detailed reporting elements for reporting of global health research that has used design**

| Item | Paper section | Detailed description | Inclusion in manuscript? Yes, No, or N/A |
| --- | --- | --- | --- |
| 1. | Title | Explicit mention of design in the title, what was designed, what process was used, intended outcomes or potential contribution. | Yes |
| 1.1 | Abstract | Statement of the health problem/design brief applied, noting whether qualitative or mixed method research (or other) was included. Abstract should contain relevant information on the health issue, design practice, methods, results and conclusions. | Yes |
| 2. | Introduction/background | Providing relevant references to the scope of the health issue, any previous work in this area or other ways of addressing the health topic; introduction of the design rationale. | Yes |
| 2.1 | Available knowledge | Literature review of past studies, knowledge and projects relevant to the problem. Statement of what is known about the problem and what has been done to address it in the past, state of the art. | Yes |
| 2.2 | Rationale for design approach | Rationale should be clearly articulated. | Yes |
| 2.3 | Description of design challenge for health | Description of design and health issue, scoping of the challenge and scale or timing at which the design was included (which may result in reframing of the initial research question). | Yes |
| 2.4 | Research aims | Explanation of the overall aims and the specific objectives of this work. | Yes |
| 3. | Methods/approach | Description of research, including design approach used and overall role of design in the work. | Yes |
| 3.1 | Theory | Description of any underlying, conceptual or motivating theories or frameworks used. | N/A |
| 3.2 | Process and timeline | Detailed description of the process or processes for applying design and research, full timeline of activities from beginning to end. | Yes |
| 3.3 | Research team characteristics and reflexivity | Description of research team and how the team addressed reflexivity.   - Geographical origin. - Discipline expertise in health, design and/or complementary disciplines and training. - Rationale for team composition. - Level of proximity to or familiarity with community/population of interest. - Prior relationship with community, wider team and/or other researchers (eg, consultants and implementing staff). - Assumptions/presuppositions regarding health topic and design. - Team attributes that could impact power and participation with community (eg, race/ethnicity, socioeconomic status and gender). | Yes |
| 3.4 | Site selection | Study setting information, including health background, geographical location, rationale and method for choosing site, and previous use of design, if any, in the setting. | Yes |
| 3.5 | Participant selection and engagement | Detailed description of participants in the design process and how the research question or intent led to inclusion; explanation of who the participants were, how they were chosen and contacted, what role they had (eg, interviewees, testers), length of involvement, extent of involvement (ie, codesigned vs consulted only), representativeness or generalisability of the participants, any sampling techniques if used or assessment of completeness of the participant group for design. | Yes |
| 3.6 | Ethical considerations | Ethics and information related to human subjects’ protection for social–behavioural research; documentation of ethics approval (if any) by a review board and participant consent, or explanation for lack thereof; addressing privacy, confidentiality and data security and internationally recognised concepts and guidelines (eg, Declaration of Helsinki and Belmont Report). | Yes |
| 3.7 | Language | Language in which the project was conducted; if in translation, credentials of translators; checking for accuracy of materials or workshop translation; familiarity of designers and participants with language and cultural nuances. | Yes |
| 3.8 | Techniques to understand (data collection tools and instruments) | Description of the types of the techniques or tools employed, rationale for method, type of data collected and any changes to methods during data collection. Description of who used research tools and with whom; should be stated separately for phases of discovery or insight gathering and testing or prototyping; what was done with this information and for what purpose it was done; any references to precedents for use. | Yes |
| 3.9 | Documentation | Statement of how documentation was carried out (eg, notes, videos, and photos), by whom, what specifically was documented (eg, interviews, affinity exercises, and observations) and role of participants in documentation. Types of data collected (eg, potentially listing and numbering of interviews, photos, videos, notes, insight statements, prototypes and pile/card sorts). | Yes |
| 3.10 | Techniques to synthesise | Explanation of process for team synthesis, analysis of collected information from the design work (eg, brainstorming and journey maps); inclusion of any data analysis software or use of large data sets to validate insights; description of team members involved in this process. | Yes |
| 3.11 | Validation approaches | Description of additional steps used to verify, validate, triangulate or test the themes emerging (eg, comparing to existing literature, expert review and feedback from participants); summary of process of reflection for researchers to understand their own role in the development of the final products and steps taken to enhance validity of the solution (other than direct testing with a small number of users). | Yes |
| 4. | Results/findings from design research and activities | Description of what was created as a result of the work: intervention, solution, policy, technology, behaviour change, service or other result; inclusion of salient features of the solution; noting how these addressed underlying health topic and what happened after design (eg, intervention or product status); documentation of ownership of what was created in the process; description of research prototypes. | Yes |
| 4.1 | Design research phase | Presentation of design research:   - Problem framing. - Design insights. - Development and refinement of insights (diverge/converge). - Description of prototyping and use of measurement/assessment and iterative refinement. - Deliverables (eg, service blueprints, maps, prototypes, storyboard). | Yes (note: prototyping was not part of this project) |
| 4.2 | Decision points | Key decision-making points and any criteria used as the design process was under way for changes made and iterations. | N/A: Prototyping and iterative testing were not part of this project so decision points were not noted. |
| 4.3 | Evidence of change or impact | Description of change or impact if any was noted, including any negative or null results; if evaluation has been done, what, if any, direct health benefits or other benefits, including social transformation, were identified and description by whom these were identified. | N/A: There was no evaluation conducted. |
| 5. | Discussion | Brief reiteration of findings or results; description of any limitations, strengths or challenges faced during research; description of how the results fit with other solutions for this health topic and how conclusions were arrived at in similar or different ways. | Yes |
| 5.1 | Design | Discussion of the transferability and specific contribution of design; description of how the solution developed is different from previous ones and fills any gap in knowledge; possible interpretation or development of a new model or methodology. | Yes |
| 6. | Conclusion | Explanation of the next key research or action to address the health topic and broader fields, noting implications for other practitioners or relevance to other contexts, commenting on the future of design for this domain. | Yes |
| 7. | Other | Description of any material interest of those involved in the research, including paid consulting.   - Statement of funding (if provided and by whom). - Description of the research team’s ongoing commitment to addressing any issues raised by participants during the research.   Contributorship   - Description statement regarding roles each author had in research and reporting. | Yes |
| 7.1 | Glossary | Any terminology, especially design terminology, which may not be understood in the lay, health, or biomedical context and vice versa. | N/A but abbreviations are included |
