# Supplementary material for: Integrating Human-Centered Design Methods Into a Health Promotion Project: Supplemental Nutrition Assistance Program Education Case Study for Intervention Design
Source: JMIR Form Res. 2023 Apr 21;7:e37515. doi: 10.2196/37515 (PMC10163394; doi:10.2196/37515)
Supplement: Multimedia Appendix 2 [file formative_v7i1e37515_app2.docx]

**Multimedia Appendix 2:** FFORC Project HCD Methods by IDEO HCD Phase*

| Inspiration | Ideation | Implementation |
| --- | --- | --- |
|  |  |  |

*Bolded methods are described in this article.

| Design Thinking Sessions | | |
| --- | --- | --- |
| Inspiration | Ideation | Implementation |
| Frame Your Design Challenge | Get Feedback | Roadmap |
| Create a Project Plan | Integrate Feedback and Iterate | Resource Assessment |
| Recruiting Tools | Find Themes | Build Partnerships |
| Secondary Research | Explore Your Hunch | Define Success |
| Expert Interview | Create Insight Statements |  |
| Define Your Audience | **Create Frameworks (Journey Map)** |  |
| **Extremes and Mainstreams** | **Get Visual (Journey Map)** |  |
| Card Sort | Brainstorm Rules |  |
|  | Brainstorm |  |
|  | How Might We |  |
|  | Bundle Ideas |  |
|  | Co-Creation |  |
|  | Download Your Learnings |  |
|  | How Might We |  |
|  | Determine What to Prototype |  |
|  | Design Principles |  |
|  | Top Five |  |
|  | Rapid Prototyping |  |

*Note: Methods are listed chronologically from top to bottom.
