# Supplementary figures and images for: Integrating Human-Centered Design Methods Into a Health Promotion Project: Supplemental Nutrition Assistance Program Education Case Study for Intervention Design
Source: JMIR Form Res. 2023 Apr 21;7:e37515. doi: 10.2196/37515 (PMC10163394; doi:10.2196/37515)

**Multimedia Appendix 3:** Meal Box Decision Tree and Intervention Options


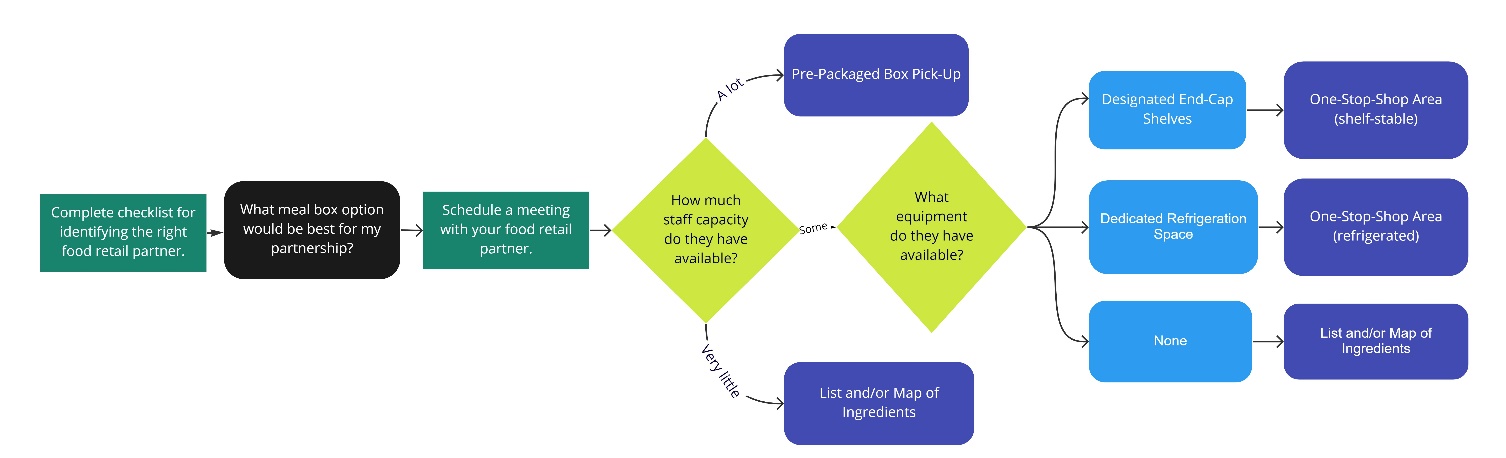

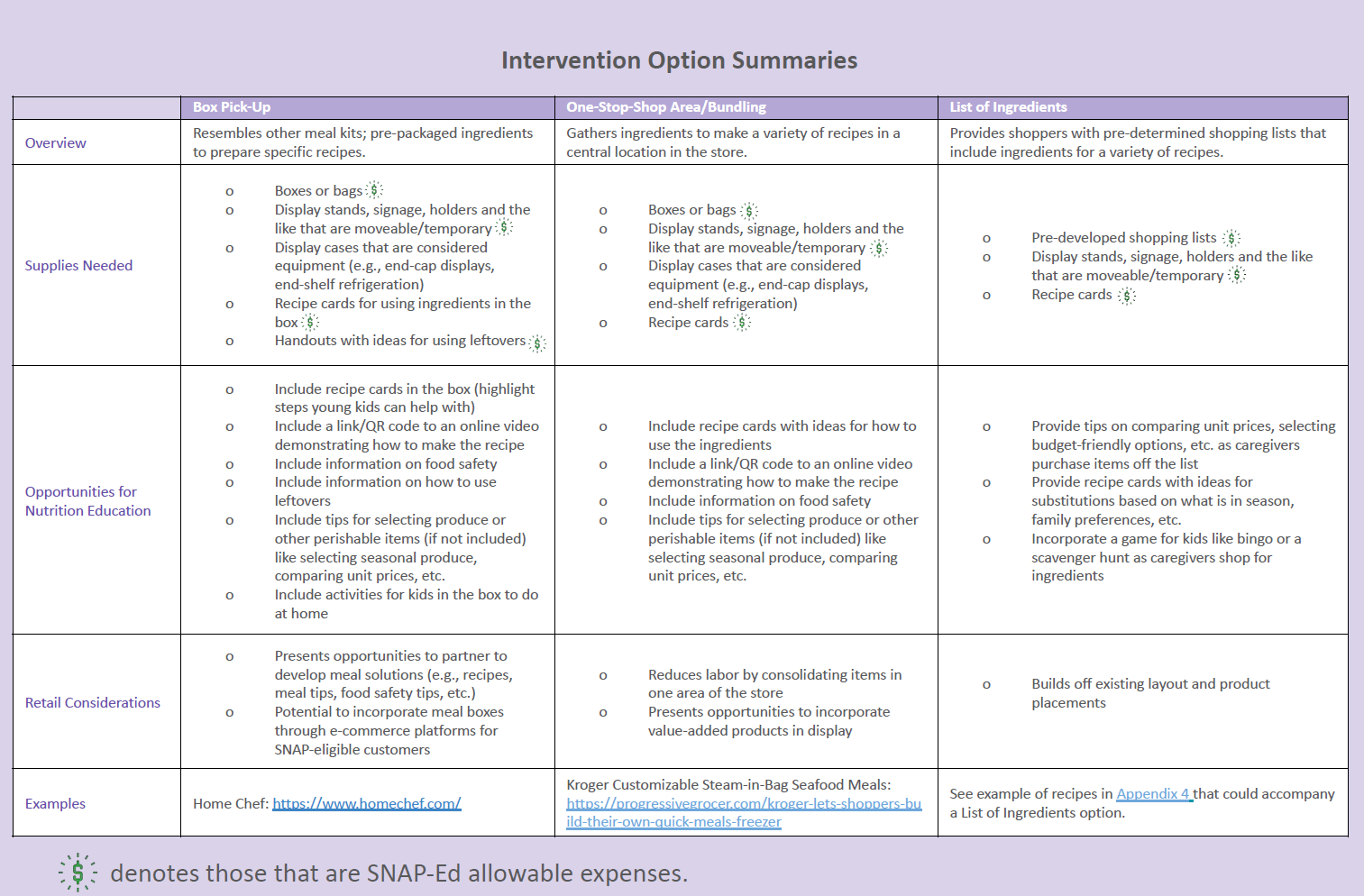

Supplement: Multimedia Appendix 3 [file formative_v7i1e37515_app3.docx]
